# *TP53* mutations predict disease control in metastatic colorectal cancer treated with cetuximab-based chemotherapy

**DOI:** 10.1038/sj.bjc.6605008

**Published:** 2009-04-14

**Authors:** A Oden-Gangloff, F Di Fiore, F Bibeau, A Lamy, G Bougeard, F Charbonnier, F Blanchard, D Tougeron, M Ychou, F Boissière, F Le Pessot, J-C Sabourin, J-J Tuech, P Michel, T Frebourg

**Affiliations:** 1 Inserm U614, Faculty of Medicine, Institute for Biomedical Research, University of Rouen, 22 Boulevard Gambetta, Rouen 76183, France; 2 Digestive Oncology Unit, Department of Hepato-Gastroenterology, Rouen University Hospital, 1 rue de Germont, Rouen Cedex 76031, France; 3 Department of Pathology, Centre Régional de Lutte Contre le Cancer Val d’Aurelle-Paul Lamarque, Parc Euromédecine, Montpellier Cedex 5 34298, France; 4 Department of Pathology, Rouen University Hospital, 1 rue de Germont, Rouen Cedex 76031, France; 5 Digestive Oncology Unit, Centre Régional de Lutte Contre le Cancer Val d’Aurelle-Paul Lamarque, Parc Euromédecine, Montpellier Cedex 5 34298, France; 6 Department of Surgery, Rouen University Hospital, 1 rue de Germont, Rouen Cedex 76031, France

**Keywords:** colorectal cancer, molecular marker, *TP53*, mutation, targeted therapy

## Abstract

Recent studies have suggested that activation of the EGFR pathway leads to malignant transformation only if the p53 protein is inactivated. Therefore, we evaluated the impact of *TP53* mutations on cetuximab-based chemotherapy (CT) sensitivity in combination with *KRAS* mutations that have been associated with cetuximab resistance. *KRAS* and *TP53* status were assessed in tumours from 64 metastatic colorectal cancer patients treated with cetuximab-based CT and correlated to clinical response using the Fisher's exact test. Times to progression (TTPs) according to gene status were calculated using the Kaplan–Meier method and compared with log-rank test. *TP53* mutations were found in 41 patients and were significantly associated with controlled disease (CD), as defined as complete response, partial response or stable disease (*P*=0.037) and higher TTP (20 *vs* 12 weeks, *P*=0.004). Remarkably, in the subgroup of 46 patients without *KRAS* mutation, but not in patients with *KRAS* mutation, *TP53* mutations were also associated with CD (*P*=0.008) and higher TTP (24 *vs* 12 weeks, *P*=0.0007). This study suggests that *TP53* mutations are predictive of cetuximab sensitivity, particularly in patients without *KRAS* mutation, and that *TP53* genotyping could have a clinical interest to select patients who should benefit from cetuximab-based CT.

In the past decade, the development of new combinations of conventional chemotherapies (CTs) and the introduction of targeted therapies have led to a dramatic improvement of the overall survival of patients with metastatic colorectal cancer (MCRC) (Meyerhardt and Mayer, 2005). Nevertheless, the variability of the response rates in MCRC patients treated with anti-EGFR in monotherapy or in association with CT underlines the urgent need of predictive markers to select the appropriate patients who can benefit from these treatments (Cunningham *et al*, 2004; Saltz *et al*, 2004; Jonker *et al*, 2007; Van Cutsem *et al*, 2007).

Anti-EGFR antibodies used in MCRC, such as cetuximab and panitumumab, are predicted to bind to the EGFR ectodomain, which prevents ligand fixation and therefore inhibits EGFR-dependent transduction cascades such as the RAS–RAF–MEK–MAPK and PIK3CA–Akt pathways (Ciardello and Tortora, 2008). From a theoretical point of view, anti-EGFR cancer treatment requires three parameters to be efficient. First, activation of the EGFR pathway should contribute to the malignant transformation. Although the mechanisms of this activation have not been clearly established in colorectal cancer (CRC), it could result from gain of copies of the *EGFR* gene, or overexpression of EGFR ligands that have both been suggested to be markers of sensitivity to anti-EGFR (Moroni *et al*, 2005; Sartore-Bianchi *et al*, 2005; Khambata-Ford *et al*, 2007; Cappuzzo *et al*, 2008a; Personeni *et al*, 2008). Second, activation of the EGFR pathway should not result from the activation of a downstream EGFR effector such as RAS or PI3KCA. Indeed several recent studies in MCRC patients have unambiguously established that the presence of a somatic *KRAS* activating mutation is highly predictive of resistance to anti-EGFR antibodies (Lièvre *et al*, 2006, 2008; Benvenuti *et al*, 2007; Di Fiore *et al*, 2007; Khambata-Ford *et al*, 2007; Amado *et al*, 2008; De Roock *et al*, 2008; Karapetis *et al*, 2008; Van Cutsem *et al*, 2008). Besides *KRAS* mutation, it has been recently reported that loss of PTEN expression/PI3KCA activation and *BRAF* mutations are also associated with resistance to anti-EGFR (Benvenuti *et al*, 2007; Frattini *et al*, 2007; Cappuzzo *et al*, 2008b; Di Nicolantonio *et al*, 2008; Jhawer *et al*, 2008; Perrone *et al*, 2009). Last, several data suggest that molecular brakes such as p53, protecting the cells against inappropriate oncogene activation should be inactivated. Indeed, recent studies have indicated that oncogenic activation of transduction cascades leads to malignant transformation, only if p53 is inactivated: alteration of the p53 pathway has been reported to be systematically observed in NSCLC with activating *EGFR* mutations suggesting that p53 inactivation is required to allow expansion of a cell with EGFR pathway activation (Mounawar *et al*, 2007). Moreover, it has been shown that activation of PIK3CA signalling activates p53-mediated growth suppression, suggesting that p53 acts as a brake for the activated PIK3CA transduction cascade (Kim *et al*, 2007).

This observation led us to hypothesise that, among MCRC without *KRAS* mutation, tumours with *TP53* mutations should be more sensitive to anti-EGFR antibodies. We therefore evaluate in this study the combined impact of *KRAS* and *TP53* status on clinical outcome in MCRC patients treated with cetuximab.

## Materials and methods

### Patients

We assessed 64 chemorefractory MCRC patients treated with cetuximab-based CT and for whom tumour tissues were available for molecular analysis. Among these patients, 44 patients had already been included in a previous study focused on the impact of *KRAS* status on the clinical response to cetuximab (Di Fiore *et al*, 2007). Tumour response was evaluated according to the response evaluation criteria in solid tumours (Therasse *et al*, 2000). Patient tumour response was classified as complete response (CR), partial response (PR), stable disease (SD) or progressive disease (PD). Patients with CR or PR or SD were considered as patients with controlled disease (CD). Follow-up was performed on clinical basis and CT scan until disease progression, death or the last follow-up at which point data were censored.

### DNA extraction

For 55 patients, DNA was extracted from paraffin-embedded tumour tissue. After macrodissection, the extraction was carried out using the DNA extraction kits from Takara (Madison, WI, USA) or Ambion (Huntingdon, Cambridgeshire, UK), according to the manufacturer's instructions. For the nine remaining patients, DNA was extracted from frozen samples using the QIAamp DNA mini kit (Qiagen, Courtaboeuf, France). For each tumour sample, the percentage of malignant cells was estimated, by morphological analysis, to at least 50%.

### *KRAS* and *TP53* genotyping

For all patients, *KRAS* mutation analysis was performed using the SNaPshot multiplex assay, as previously described (Di Fiore *et al*, 2007). *TP53* exons 5–8 were PCR amplified from tumour DNA (primer sequences are available upon request), and after purification using the NucleoSpin Extract II kits (Masherey Nagel, Düren, Germany), PCR products were sequenced using the BigDye Terminator v3.1 kit (Applied Biosystems, Foster City, CA, USA) and a 3130*xl* Genetic Analyzer (Applied Biosystems). For nine patients, DNA was extracted from frozen tissue allowing the screening of mutations by high resolution melting analysis using the LightScanner instrument from Idaho Technology (Salt Lake City, UT, USA). For these nine patients, only the amplicons with an aberrant denaturation curve were sequenced. For the 55 remaining samples, DNA was extracted from paraffin-embedded tumour tissue and *TP53* mutations were detected by direct sequencing. Considering the presence of non-malignant cells in tumour samples, the presence of a *TP53* mutation in the tumour was defined as the appearance of a mutant peak with a height of at least 25% of the wild type, and each detected *TP53* mutation was confirmed by a second sequencing analysis performed on an independent PCR. For both *KRAS* and *TP53* mutational analyses, data were analysed without knowing the clinical response of patients.

### Statistical analysis

Response to treatment according to the mutational status was evaluated using the Fisher's exact test. The time to progression (TTP) was calculated as the period from the beginning of treatment to the first observation of disease progression or to death or the last follow-up at which point data were censored. The TTPs were estimated using the Kaplan–Meier method and compared with the log-rank test. Multivariate analysis of predictive factors of TTP was performed using a Cox regression model with calculation of hazard ratio (HR) and a confidence interval (CI) of 95%. A *P*-value ⩽0.05 was considered to indicate statistical significance. All statistics were calculated using the StatView statistical software (SAS Institute Inc., Cary, NC, USA).

## Results

### Patient characteristics and outcome

A total of 64 chemorefractory MCRC patients treated with cetuximab-based CT, including 45 men and 19 women with a mean age of 59.5 years (range 20–82; s.d. 12.8), were included in this study (Table 1). Patients had received a mean of 1.9 previous metastatic CT lines before cetuximab and 90% of them were irinotecan refractory. Sixty-two patients received cetuximab with irinotecan-based CT, one received cetuximab with combined irinotecan and oxaliplatin-based CT and one received cetuximab alone. Response to cetuximab-based CT showed that 39 patients (61%) had a CD (2 CR, 14 PR and 23 SD) whereas 25 were in PD (39%). The median TTP was 24 weeks in patients with CD *vs* 12 weeks in patients with PD (*P*<0.0001).

### *KRAS* status and clinical outcome

A *KRAS* mutation was found in 18 patients (28%). As presented in Table 2, the three most frequent mutations were c.35G>T, c.38G>A and c.35G>A. None of the 16 patients with CR or PR had a *KRAS* mutation. In contrast, 7 out of 23 (30%) patients with SD and 11 out of 25 (44%) with PD had a *KRAS* mutation respectively. Using the Fisher's exact test, we found that *KRAS* mutations were significantly associated with PD *vs* CD (*P*=0.044). The median TTP of patients with *KRAS* mutation was significantly lower as compared to those with wild-type *KRAS* (12 *vs* 20 weeks, *P*=0.034).

### *TP53* mutational status and clinical outcome

A total of 46 *TP53* mutations were found in 41 out of 64 patients (64%). Recurrent mutations were found at codons 152, 175, 213, 245, 248, 273 and 282 as presented in Table 2. No significant difference was found for the main clinical characteristics between patients with and without *TP53* mutation (Table 1). A *TP53* mutation was detected in 29 out of 39 patients with CD (74%) and in 12 out of 25 patients with PD (48%). *TP53* mutations were significantly associated with CD *vs* PD (*P*=0.037; Table 3). Moreover, median TTP in patients with *TP53* mutation was significantly increased as compared to patients without detectable *TP53* mutation (20 *vs* 12 weeks, *P*=0.004).

### Combined *KRAS* and *TP53* status and clinical outcome

**Figure 1:**
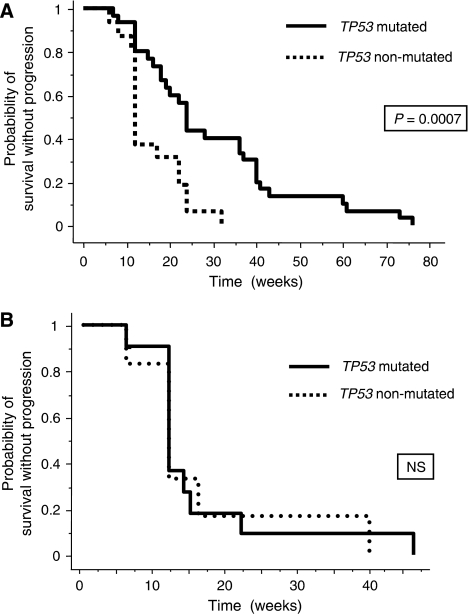
Time to progression curves of MCRC patients treated with cetuximab according to the *TP53* genotype. (**A**) Patients without detectable *KRAS* mutation. (**B**) Patients with *KRAS* mutation.

Considering the hypothesis of this study, we then focused our analysis on the subgroup of 46 patients without *KRAS* mutation. In this subgroup, we detected a *TP53* mutation in 30 patients (65.2%; Table 4). The main clinical characteristics between patients with and without *TP53* mutation were not significantly different (Table 1). A *TP53* mutation was found in 25 out of 32 (78%) patients with CD as compared to 5 out of 14 (36%) with PD (*P*=0.008; Table 4). In patients with wild-type *KRAS*, those having *TP53* mutation had a significantly higher TTP as compared to those without detectable *TP53* mutation (24 *vs* 12 weeks, *P*=0.0007; Figure 1A). In contrast, in the subgroup of patients with *KRAS* mutation, the median TTPs were not different between patients with and without *TP53* mutation (Figure 1B).

### Multivariate analysis

A Cox regression model was performed to determine the predictive factor of TTP in the whole population. This analysis included the following variables: sex, age ⩾70 years, previous metastatic CT lines >2, *KRAS* and *TP53* status. *TP53* mutations and *KRAS* mutations were identified as two independent predictive factors (HR=1.99, 95% CI 1.09–3.63, *P*=0.024 and HR=0.48, 95% CI 0.25–0.94, *P*=0.032 respectively).

## Discussion

*KRAS* mutation has been reported in several studies as a predictive marker of anti-EGFR resistance in MCRC (Lièvre *et al*, 2006, 2008; Benvenuti *et al*, 2007; Di Fiore *et al*, 2007; Khambata-Ford *et al*, 2007; Amado *et al*, 2008; De Roock *et al*, 2008; Karapetis *et al*, 2008; Van cutsem *et al*, 2008) and the characterisation of other parameters underlying the response variability to anti-EGFR is now an important issue. Investigation in this MCRC patients series of other markers, which had previously been shown to be associated either to sensitivity or resistance to anti-EGFR antibodies, revealed a *BRAF* mutation in 2 out of 49 (4%) patients (these patients having no detectable *KRAS* mutation and presenting an SD), a *PIK3CA* mutation in 5 out of 45 (11%) patients (2 out of 27 patients with CD and 3 out of 18 patients with PD) and an *EGFR* gene copy number increase, as defined by a number of *EGFR* per nucleus above 2.5 in 40% of the cells, in 9 out of 47 (19%) tumours (2 tumours with CR, 2 with PR and 3 being stabilised and the last one, which progressed under cetuximab-based therapy, was found to have a *KRAS* mutation). Although the frequency of these alterations is in agreement with the published studies (Moroni *et al*, 2005; Sartore-Bianchi *et al*, 2005; Lièvre *et al*, 2006, 2008; Benvenuti *et al*, 2007; Khambata-Ford *et al*, 2007; Cappuzzo *et al*, 2008a, 2008b; Di Nicolantonio *et al*, 2008; Personeni *et al*, 2008; Perrone *et al*, 2009), indicating that our series is representative of MCRC, these alterations did not appear statistically associated with clinical outcome to cetuximab-based CT, considering the sample size. In contrast, this study, performed in 64 MCRC patients, suggests that *TP53* mutations are predictive markers of cetuximab sensitivity, particularly in the subgroup of patients without detectable *KRAS* mutation. Indeed, our results showed that *TP53* mutations, in patients with wild-type *KRAS*, were associated with a higher CD and TTP (Table 4; Figure 1A), and the multivariate analysis suggested that both *TP53* and *KRAS* mutations were independent predictive markers. Considering that alterations of *BRAF* and *PIK3CA/PTEN* have been shown to result also in resistance to anti-EGFR antibodies (Benvenuti *et al*, 2007; Frattini *et al*, 2007; Cappuzzo *et al*, 2008b; Di Nicolantonio *et al*, 2008; Jhawer *et al*, 2008; Perrone *et al*, 2009), we analysed the value of *TP53* mutation in the group of patients without detectable mutations within *KRAS*, *BRAF* and *PIK3CA*. Among the 46 MCRC patients without detectable *KRAS* mutation, we could analyse these genes in 30 patients for whom sufficient tumour DNA was available, and 24 of them had no detectable mutation of *BRAF* and *PIK3CA*. In the subgroup of 24 patients without detectable mutation within *KRAS*, *BRAF* and *PIK3CA*, we observed, in this small sample, a trend but not significant difference between the group of patients with (18) and without (6) *TP53* mutation, in term of CD (a *TP53* mutation was found in 15 out of 19 (79%) patients with CD as compared to 3 out of 5 (60%) with PD, *P*=0.568) and TTP (20 *vs* 12 weeks, *P*=0.0931).

The association that we report between *TP53* mutations and better clinical outcome may appear unexpected because, in CRC, most of studies have shown that *TP53* mutations are associated with a worse prognosis in stage II–III CRC patients (Westra *et al*, 2005). However, the predictive function of *TP53* mutations in MCRC patients treated with targeted therapies has not been so far established. Indeed, the only previous study performed on MCRC patients, which had evaluated the predictive value of *TP53* mutations in the context of targeted therapies, concerns the anti-vascular epidermal growth factor antibody bevacizumab, and no correlation has been found between the *TP53* status and the clinical response (Ince *et al*, 2005). Considering that 63 out of 64 patients received irinotecan in combination with cetuximab in our study, we cannot formally exclude that the *TP53* status might specifically influence the response to the conventional CT. Despite the absence of control group in our work, this hypothesis seems unlikely because it has been suggested in cellular models that *TP53* status does not modulate the response to irinotecan (McDermott *et al*, 2005).

In contrast, a recent study performed in cellular models has suggested that *TP53* status may influence the response to targeted therapies (Kim *et al*, 2007). In a normal cell, the p53 protein acts not only as a guardian of the genome, which is activated when DNA damage occurs, but also as a policeman of oncogenes, which becomes active when oncogenes are inappropriately activated, and this activation induces apoptosis and/or senescence (Efeyan and Serrano, 2007; Halazonetis *et al*, 2008). Moreover, alteration of the p53 pathway has been reported to be observed in NSCLC with activating *EGFR* mutations suggesting that p53 inactivation is required to allow expansion of a cell with EGFR pathway activation (Mounawar *et al*, 2007). Supporting this assumption, we found that 8 out of 9 (89%) tumours with an *EGFR* copy number increase harboured a *TP53* mutation whereas a *TP53* mutation was found in 22 out of 38 (56%) tumours without detectable *EGFR* copy number increase. Finally, it has been shown that p53-mediated growth suppression is induced by PIK3CA signalling activation suggesting that p53 acts as a brake for the PIK3CA transduction cascade (Kim *et al*, 2007). Therefore, it is likely to speculate that activation of the EGFR pathway will contribute to cancer and that anti-EGFR antibodies will be efficient on tumour, only if p53 is inactivated. This hypothesis is supported by our results showing that CD and TTP were significantly increased in patients with *TP53* mutation treated with cetuximab-based CT. Recently, it has been shown in cellular models that loss of p53 results into an *EGFR* promoter induction (Bheda *et al*, 2008). Therefore, our results might be explained not only by the fact that activation of EGFR is oncogenic only if *TP53* is inactivated, but also by the fact that inactivation of *TP53* could be one of the mechanisms leading to EGFR activation.

In conclusion, our study suggests that *TP53* genotyping could have an additional value in MCRC patients without *KRAS* mutation to optimise the selection of patients who should benefit from anti-EGFR therapies. The relationship between *TP53* status and sensitivity to anti-EGFR should be investigated in cellular models and the clinical relevance of our results should be confirmed on larger MCRC series.

## Figures and Tables

**Table 1:** Patients characteristics according to their *TP53* and *KRAS* mutational status

	**All (*n*=64)**			***KRAS* non-mutated (*n*=46)**		
	***TP53***			***TP53***		
	**Mutated**	**Non-mutated**	***P***	**Mutated**	**Non-mutated**	***P***
Sex ratio (men/women)	2.25	2	NS	2.22	1.8	NS
Age ⩾70 years (%)	24.4	23.8	NS	26.7	28.6	NS
Irinotecan-refractory patients (%)	90	90	NS	93	100	NS
>2 previous metastatic CT lines (%)	10	10	NS	13.8	7.7	NS
Mean of previous metastatic CT lines	1.91	1.92	NS	1.92	1.91	NS

CT=chemotherapy; NS=not significant.

**Table 2:** Distribution of *KRAS* and *TP53* mutations

**Mutation**	**Predicted effect on protein**	**Location**	**Number of samples^a^**
*KRAS mutations*			
c.34G>T	p.G12C	Exon 2	2
c.34G>A	P.G12S	Exon 2	1
c.35G>T	p.G12V	Exon 2	5
c.35G>A	p.G12D	Exon 2	3
c.35G>C	p.G12A	Exon 2	1
c.37G>T	p.G13C	Exon 2	1
c.38G>A	p.G13D	Exon 2	5
			
*TP53 mutations*			
c.379T>C	p.Ser127Pro	Exon 5	1
c.382_423del	p.Ala129_Pro142del	Exon 5	1
c.404G>T	p.Cys135Phe	Exon 5	1
c.449_461del	p.Pro152AlafsX14	Exon 5	1
c.455C>T	p.Pro152Leu	Exon 5	2
c.455dup	p.Pro153AlafsX28	Exon 5	1
c.458C>T	p.Pro153Leu	Exon 5	1
c.463A>T +c.464C>T	Unknown	Exon 5	1
c.470T>A	p.Val157Asp	Exon 5	1
c.524G>A	p.Arg175His	Exon 5	4
c.527G>A	p.Cys176Tyr	Exon 5	1
c.588_609dup	p.Glu204SerfsX12	Exon 6	1
c.614A>G	p.Tyr205Cys	Exon 6	1
c.637C>T	p.Arg213X	Exon 6	2
c.638G>T	p.Arg213Leu	Exon 6	1
c.659A>G	p.Tyr220Cys	Exon 6	2
c.685_686del	p.Cys229TyrfsX10	Exon 7	1
c.713G>A	p.Cys238Tyr	Exon 7	1
c.722C>A	p.Ser241Tyr	Exon 7	1
c.724T>C	p.Cys242Arg	Exon 7	1
c.731G>A	p.Gly244Asp	Exon 7	1
c.733G>A	p.Gly245Ser	Exon 7	2
c.734G>A	p.Gly245Asp	Exon 7	1
c.743G>A	p.Arg248Gln	Exon 7	4
c.763A>T	p.Ile255Phe	Exon 7	1
c.705_713dup	p.Tyr236_Cys238dup	Exon 7	1
c.790_791del	p.Leu264ThrfsX7	Exon 8	1
c.796G>A	p.Gly266Arg	Exon 8	1
c.817C>T	p.Arg273Cys	Exon 8	2
c.818G>A	p.Arg273His	Exon 8	2
c.824G>A	p.Cys275Tyr	Exon 8	1
c.844C>T	p.Arg282Trp	Exon 8	2
c.892G>T	p.Gly298X	Exon 8	1

a Number of samples indicates the number of tumour samples in which each mutation was found.

**Table 3:** Clinical response to cetuximab according to the *TP53* status in 64 MCRC patients treated with cetuximab

	**Controlled disease**			
	**Complete response**	**Partial response**	**Stable disease**	**Progressive disease**
*TP53* non-mutated	0	4	6	13
*TP53* mutated	2	10	17	12

*Note*: the *TP53* status was assessed by sequencing analysis between exons 5 and 8.

*P*=0.037 for *TP53* mutations and CD *vs* PD.

**Table 4:** Clinical response to cetuximab according to the *TP53* status in the 46 MCRC patients without detectable *KRAS* mutation treated with cetuximab

	**Controlled disease**			
	**Complete response**	**Partial response**	**Stable disease**	**Progressive disease**
*TP53* non-mutated	0	4	3	9
*TP53* mutated	2	10	13	5

*Note*: the *TP53* status was assessed by sequencing analysis between exons 5 and 8.

*P*=0.008 for *TP53* mutations and CD *vs* PD.
